# A Molecularly Imprinted Polymer Nanobodies (nanoMIPs)-Based Electrochemical Sensor for the Detection of *Staphylococcus epidermidis*

**DOI:** 10.3390/s25072150

**Published:** 2025-03-28

**Authors:** Witsanu Rapichai, Chularat Hlaoperm, Adriana Feldner, Julia Völkle, Kiattawee Choowongkomon, Jatuporn Rattanasrisomporn, Peter A. Lieberzeit

**Affiliations:** 1 Department of Companion Animal Clinical Sciences, Faculty of Veterinary Medicine, Kasetsart University, Bangkok 10900, Thailand; tswitsanu@gmail.com (W.R.); chularat.h@ku.th (C.H.); 2 Department of Biochemistry, Faculty of Science, Kasetsart University, Bangkok 10900, Thailand; kiattawee.c@ku.th; 3 University of Vienna, Faculty for Chemistry, Department of Physical Chemistry, Waehringer Strasse 42, A-1090 Vienna, Austria; adriana.feldner@gmx.at (A.F.); julia.voelkle@univie.ac.at (J.V.); 4 University of Vienna, Faculty for Chemistry, University of Vienna, Waehringer Strasse 42, A-1090 Vienna, Austria

**Keywords:** molecularly imprinted polymers, nanobody, electrochemical impedance spectroscopy, *Staphylococcus epidermidis*

## Abstract

**Highlights:**

This study presents a novel electrochemical sensor utilizing molecularly imprinted polymer nanobodies (nanoMIPs) for ultrasensitive and selective detection of *Staphylococcus epidermidis*. The sensor integrates nanoMIPs synthesized via solid-phase imprinting of whole bacteria onto gold screen-printed electrodes (AuSPE) functionalized with a self-assembled monolayer (SAM) of 16-mercaptohexadecanoic acid (MHDA). Electrochemical impedance spectroscopy (EIS) demonstrated a remarkable detection limit of 1 CFU/mL and a linear range of 10^1^–10^6^ CFU/mL, outperforming conventional PCR, real-time PCR, and other biosensors. Selective binding was confirmed against *E. coli* and *B. subtilis*, validated by SEM/AFM characterization showing increased surface roughness and particle immobilization. The platform offers cost-effectiveness, rapid analysis (<30 min), and disposability, making it promising for real-time monitoring of biofilm-associated infections on medical devices, with potential applications in clinical diagnostics and infection control.

**What are the main findings?**

- A nanoMIP-based electrochemical sensor was developed for detecting *Staphylococcus epidermidis*, achieving high specificity and sensitivity with a detection limit as low as 1 CFU/mL.
- This sensor demonstrates potential for application in medical diagnostics and contamination monitoring due to its simplicity, cost-effectiveness, and reliable performance.

**What is the implication of the main finding?**

- The main finding of this study demonstrates that the developed nanoMIP-based electrochemical sensor effectively detects *Staphylococcus epidermidis* with high selectivity and an impressively low detection limit of 1 CFU/mL.
- This result highlights the sensor’s potential for application in medical diagnostics and safety monitoring of medical devices, addressing a critical need for rapid and precise bacterial detection.

**Abstract:**

Methicillin-resistant *Staphylococcus epidermidis* (MRSE) contamination is commonly found on human skin and medical devices. Herein, we present a sensor utilizing molecularly imprinted polymer nanobodies (nanoMIP) for recognition and electrochemical impedance spectroscopy (EIS) to detect *S. epidermidis.* Sensor manufacturing involves synthesizing nanoMIP via solid-phase synthesis using whole bacteria as templates. Screen-printed gold electrode (AuSPE)-modified 16-mercaptohexadecanoic acid (MHDA) served to immobilize the nanoMIPs on the sensor surface through an amide bond, with the remaining functional groups blocked by ethanolamine (ETA). Scanning electron microscope (SEM) analysis of the modified AuSPE surface reveals immobilized spherical nanoMIP particles of 114–120 nm diameter, while atomic force microscope (AFM) analysis showed increased roughness and height compared to bare AuSPE. The sensor is selective for *S. epidermidis*, with a remarkable detection limit of 1 CFU/mL. This research demonstrates that the developed nanoMIP-based sensor effectively detects *S. epidermidis*. Further research will focus on developing protocols to integrate the nanoMIP-based EIS sensor into medical and industrial applications, ultimately contributing to improved safety for both humans and animals in the future.

## 1. Introduction

*Staphylococcus epidermidis* is a bacterium belonging to the family *Staphylococcaceae*. It is catalase positive and oxidase negative, commonly found in cell clusters. *S. epidermidis* is a Gram-positive, spherical bacterium that arranges in grape-like clusters. It is non-motile, non-spore-forming, and classified as a facultative anaerobe, capable of growing in both oxygen-rich and oxygen-deprived environments. It produces white colonies on culture media. *S*. *epidermidis* includes both coagulase-positive and coagulase-negative strains, with the coagulase-negative group being the primary cause of pathogenicity. It is a significant component of the normal microbiota, predominantly found on the human skin and mucous membranes [1,2,3,4]. Recent epidemiological data demonstrate that *S. epidermidis* remains a significant nosocomial pathogen, particularly in association with invasive medical devices including endotracheal tubes, prosthetic cardiac valves, artificial joints, and intravascular catheters. The increasing prevalence of *S. epidermidis* infections correlates with the expanded use of these implantable medical devices, establishing this organism as one of the predominant etiological agents of healthcare-associated infections [5,6,7]. The attachment of *S. epidermidis* to medical devices, followed by their proliferation and development of biofilm matrices, frequently results in nosocomial infections and compromised device functionality [8]. Moreover, infectious *S. epidermidis* can disseminate from the primary colonization site to establish secondary infections in other physiologically permissive environments. Conventional antimicrobial therapies demonstrate minimal to negligible efficacy against established biofilm communities on colonized medical devices. Therefore, surgery to extract and replace the device in question is frequently indicated. In cases where surgical intervention is contraindicated, patients require chronic suppressive antibiotic therapy for their entire lives, contributing to significant morbidity and mortality [9,10]. No commercially available vaccine exists for preventing *S*. *epidermidis* infections. Consequently, rapid and accurate detection methods are crucial for clinical prevention strategies and play a vital role in mitigating healthcare-associated infections (HAIs).

Current diagnostic methodologies to detect *S. epidermidis* in clinical settings encompass several approaches: direct microscopic examination of Gram-stained specimens, microbiological cultivation techniques, immunoassay-based detection methods, and automated phenotypic identification systems. However, each methodology presents distinct limitations. Direct microscopic examination, while representing the most fundamental bacteriological diagnostic technique, leads to both poor analytical sensitivity and inadequate taxonomic specificity. Although microbiological cultivation remains the predominant method for identifying infectious agents in clinical diagnostics, its extensive incubation requirements preclude rapid pathogen identification, with typical time-to-result intervals spanning 24–72 h [11,12]. In certain immunological assays, elevated ion concentrations during sample preparation or in buffers may destabilize or partly denature bacterial surface antigens, such as polysaccharide intercellular adhesin and other biofilm-associated proteins, or interfere with antibody binding. This may result in false-negative outcomes [13]. Recent advances in analytical instrumentation have yielded automated detection and identification systems; however, their applications remain constrained by both narrow scope and prohibitive costs, resulting in limited implementation. Molecular diagnostic assays are widely applied in clinical diagnostics and research, because they are straightforward to operate, have rapid turnaround time, and superior analytical sensitivity and specificity. However, they come at comparably high costs [13,14,15,16].

Molecularly imprinted polymers (MIPs) are materials with specific binding sites for target analytes. These MIPs selectively bind to molecules that match the geometric configuration of the imprinted cavities on their surface, while non-specific physical interactions are minimized. The synthesis of MIPs involves using a target analyte as a template molecule, which is mixed with functional monomers to create the molecular imprint. After polymerization and subsequent template removal through appropriate extraction methods, the resulting polymer contains specific recognition sites for the target molecule. The polymerization process involves the reaction between monomers and crosslinkers directed by the template molecule. Upon completion of the reaction, the template is extracted using suitable solvents, yielding a polymer with specific binding cavities that can effectively recognize and bind to it [17,18,19,20,21,22,23,24,25,26].

Electroanalytical techniques constitute a fundamental domain within analytical chemistry, enabling quantitative and qualitative analysis of specific analytes with electrochemical systems. These rely on measuring electrochemical parameters—usually current or potential—at electrode–solution interfaces and yield output depending on analyte concentration. Compared to alternative analytical strategies, such as chromatography or spectroscopy, electroanalytical methods offer distinct advantages in terms of simplification, miniaturization potential, and cost-effectiveness, facilitating rapid and precise detection protocols. Among these methods, electrochemical impedance spectroscopy (EIS) represents a particularly significant technique that quantifies circuit impedance in ohmic units. EIS demonstrates several key advantages over other electrochemical approaches, primarily due to its steady-state operational characteristics, the possibility to analyze small signals, and using a wide frequency range. State-of-the art electrochemical workstations enable EIS measurements across frequencies spanning from sub-millihertz to Megahertz ranges, providing comprehensive electrochemical characterization capabilities [27,28,29]. The fundamental principle of EIS lies in measuring electrical impedance, which represents the resistance of an electrical circuit to the flow of alternating current (AC) generated by applying a small alternating voltage (AV). EIS can be conducted either with or without a redox probe, such as potassium ferricyanide/ferrocyanide ([Fe(CN)₆]³⁻/⁴⁻), added to the solution. In the presence of a redox probe, a Faradaic current is generated, resulting in a Faradaic EIS sensor. At such conditions, the charge transfer resistance (R_ct_) is typically influenced by processes occurring at the electrode surface, including interactions such as the binding of a receptor with its target [30,31,32,33,34].

nanoMIPs are considered optimal receptor candidates for sensors designed to function at unpredictable environmental conditions. These may include the presence of denaturing agents and degradative enzymes capable of compromising the stability of bio-derived receptors such as proteins, antibodies, and aptamers. Hlaoperm et al. (2023) [35] successfully synthesized molecularly imprinted polymer nanoparticles with high specificity for *Staphylococcus epidermidis*. This achievement highlights their suitability for detecting this bacterium [35]. This research aims to integrate nanoMIPs developed in previous work into electrochemical techniques, representing the first study to establish a method for detecting *S*. *epidermidis* aiming at detecting it in real-world settings.

The novelty of this work lies in the development of a pioneering electrochemical sensor that integrates nanoMIPs and AuSPE for the ultrasensitive and selective detection of *S*. *epidermidis*. Unlike traditional methods such as PCR and real-time PCR, which are costly and time-consuming, this sensor achieves an unprecedented detection limit of 1 CFU/mL and a rapid analysis time of less than 30 min, leveraging solid-phase imprinting of whole bacteria and EIS. This innovative approach does not only outperform existing biosensors in sensitivity and specificity—demonstrated against *Escherichia coli* and *Bacillus subtilis*—but also offers a cost-effective, disposable platform with potential for real-time monitoring of biofilm-associated infections on medical devices, marking a significant advancement in clinical diagnostics and infection control.

## 2. Materials and Methods

### 2.1. Chemicals and Materials

Potassium ferricyanide (K_3_Fe(CN)_6_), potassium ferrocyanide (K_4_Fe(CN)_6_), N-tert-butylacrylamide (TBAM) and ammonium persulfate 98% extra pure were purchased from Thermo Fisher Scientific Inc. (Viehmarktgasse, Wien, Austria). Potassium chloride (KCl) was purchased from VWR Chemicals (VWR International BV, Geldenaaksebaan, Belgium). Sodium chloride was purchased from AppliChem (Ottoweg, Darmstadt, Germany). Glutaraldehyde (50% in H_2_O), 16-mercaptohexadecanoic acid (MHDA), N-hydroxysuccinimide (NHS), and ethanolamine (ETA) were purchased from Sigma-Aldrich (Merck Gesellschaft mbH, Vienna, Austria). 1-Ethyl-3-(3-dimethylaminopropyl) carbodiimide hydrochloride (EDC) were ordered from abcr GmbH (Im Schlehert, Karlsruhe, Germany).

### 2.2. Bacteria Cultivation

Three bacteria strains (*Staphylococcus epidermidis*, *Escherichia coli* and *Bacillus subtilis*) were cultured freshly for 24 h at 37 °C in lysogeny broth containing 10 g/L proteose peptone, 5 g/L NaCl, 1 g/L D-glucose monohydrate, and 5 g/L yeast extract. Following incubation, the cell suspensions were centrifuged at 1900× *g* for 10 min and washed twice with autoclaved distilled water at sterile conditions prior to subsequent use.

### 2.3. Immobilization of S. epidermidis on Glass Slide

**Figure 1:**
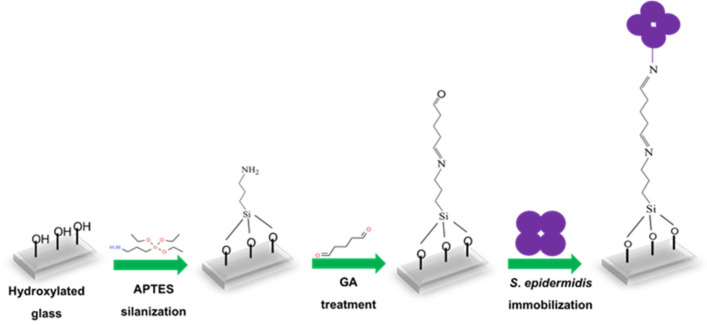
Schematic representation of *S. epidermidis* immobilization on glass slide.

Glass slides (approximately 1.5 × 1.5 cm) were initially cleaned in ethanol using an ultrasonic bath for 10 min, followed by incubation in a vacuum plasma cleaner (Diener electronic GmbH & Co. KG, Ebhausen, Germany) for 15 min to generate –OH groups on the surface. Figure 1 shows the steps leading to immobilized *S*. *epidermidis*. The activated slides were incubated with 0.05% 3-aminopropyl-triethoxysilane (APTES) in anhydrous toluene in the dark at room temperature for 2 h and subsequently washed with anhydrous toluene. They were then immersed in 5 mM glutaraldehyde (GA) and incubated for 1 h, followed by washing two times with distilled water to remove excess GA. *S*. *epidermidis* bacterial suspension (150 µL) was added to the center of the modified glass slides and incubated at 37 °C overnight. The following day, non-immobilized bacteria were removed by washing the slides five times with water, after which they were left to dry. Finally, the unreacted linker sites were blocked by treating them with 10 mM ethanolamine (ETA) for 30 min at room temperature in the dark, followed by washing five times with water.

### 2.4. MIP Nanobody (nanoMIP) Synthesis

**Figure 2:**
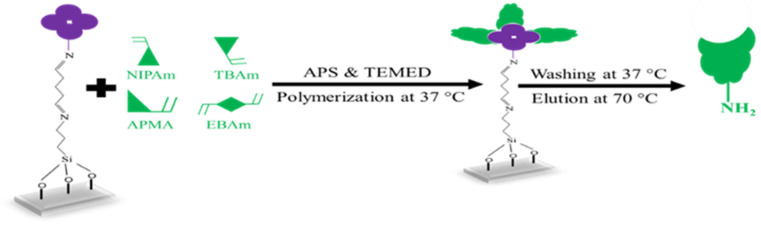
Schematic representation of the nanoMIPs fabrication.

Three distinct monomers were utilized, namely, N-isopropyl acrylamide (NIPAm, 61 mg; 0.54 mmol), N-tert-butylacrylamide (TBAm, 7.7 mg; 0.06 mmol), and N-(3-aminopropyl) methacrylamide hydrochloride (APMA, 10.7 mg; 0.06 mmol) together with ethylene bisacrylamide (EBAm, 5.6 mg; 0.06 mmol) as the crosslinker. A volume of 0.5 mL of each monomer and crosslinker solution was mixed with 500 µL ethanol and 13 mL distilled water in a centrifuging vial and sonicated for 10 min. Figure 2 sketches the synthesis steps leading to nanoMIPs: the solution was flushed with Argon to remove oxygen. The glass slides containing immobilized *S*. *epidermidis* were then placed into glass Petri dishes and covered by adding approximately 15 mL of the monomer solution. Subsequently, N,N,N′,N′-Tetramethylethylenediamine (TEMED; 200 µL in 800 µL ethanol) and ammonium persulfate (APS; 200 mg in 800 µL water) were added until the solution became opaque, after which polymerization was allowed to proceed at 37 °C for 3 h. To extract nanoMIP particles, the immobilized glass slides were washed 3 times with distilled water at a temperature of 37 °C. After removing low-affinity nanoMIPs, immobilized glass slides were washed 2 times with 50 mL distilled water at 70 °C. High-affinity nanoMIPs were evaporated to ~2 mL, filtered through a 2.5 µm membrane filter, and stored at 4 °C until use.

### 2.5. Gold Electrode Modification and Characterization

**Figure 3:**
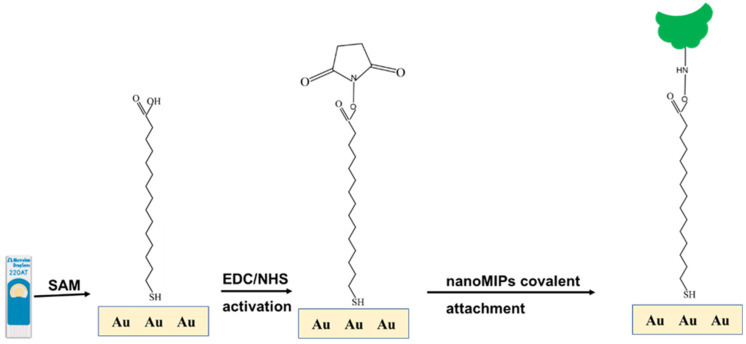
Schematic representation of the self-assembled nanoMIP fabrication step.

Bare gold screen-printed electrode (AuSPE, DRP 220 AT, Metrohm Inula GmbH, Vienna, Austria) was washed with ethanol and dried in air stream. AuSPE was pretreated by dropping 100 µL 0.1 M NaCl for 5 min. Figure 3 shows the stepwise fabrication of the modified AuSPE. First, bare AuSPE was immersed in an ethanolic solution 16-mercaptohexadecanoic acid (16-MHDA) at room temperature overnight. The resulting Au/16-MHDA electrode was rinsed with ethanol several times, and dried in an air steam. At the working electrode, 10 µL of 0.4 M N-(3-Dimethylaminopropyl)-N′-ethylcarbodiimide/0.1 M N-hydroxysuccinimide (EDC/NHS) coupling solution in 1X PBS was dropped for 10 min to generate N-hydroxysuccinimide ester, rinsed with distilled water and dried with air stream. The modified electrode surface was then treated with 10 µL nanoMIP solution for 1 h, rinsed with distilled water, and dried in air stream. Unreacted activated carboxylic groups were then blocked with 10 mM ethanolamine (ETA) for 10 min, rinsed with distilled water, and dried as before. Each step was assessed by electrochemical measurements. The nanoMIP-based AuSPE was detected by atomic force microscopy (AFM) and scanning electron microscope (SEM).

### 2.6. Electrical Measurement

Electrochemical impedance spectroscopy (EIS) measurements took place using the µStat-i 400 portable bipotentiostat/galvanostat/impedance analyzer (Metrohm Inula GmbH, Vienna, Austria) using 10 mM K_4_[Fe(CN)_6_]/K_3_[Fe(CN)_6_] in 0.1 M KCl solution as the redox indicator. After incubation with the bacteria solutions, the modified AuSPE were gently washed three times with distilled water to remove any unbound bacteria. The DropSens EIS analyzer was connected to a PC using Dropview as analytical software (Metrohm Inula GmbH, Vienna, Austria). To reduce noise, the Faraday cage was employed for all electrochemical experiments. Impedance analysis took place at an amplitude (E_amp_) of 10 mV at E_dc_ of 0.2 V from 0.1 Hz to 100 kHz. Experimental results were fitted to a Randles equivalent circuit using the DropView 8400 software (Metrohm Inula GmbH, Vienna, Austria).

### 2.7. Selectivity and Sensitivity

To determine the selectivity of the nanoMIP-based AuSPE sensor, tests were conducted with *S. epidermidis*, *B*. *subtilis*, and *E*. *coli* at identical concentrations of 10^1^ CFU/mL using sterile phosphate-buffered saline (1X PBS, pH 7.4) as diluent. A droplet of each bacterial solution was applied to the surface of the working electrode (WE) for 30 min, followed by EIS measurement. All experiments were performed in triplicate, followed by recording changes in R_ct_ values. To determine the sensitivity and limit of detection (LOD) of the sensor, *S. epidermidis* suspensions ranging from 10^1^ to 10^6^ CFU/mL in 1X PBS, pH 7.4 were tested, with each concentration incubated on the electrode surface for 30 min, followed by electrochemical impedance spectroscopy (EIS) measurements using 10 mM K₄[Fe(CN)₆]/K₃[Fe(CN)₆] in 0.1 M KCl as the redox probe. Changes in charge transfer resistance (R_ct_) were recorded in triplicate. The sensor characteristic resulted from plotting R_ct_ against the logarithmic bacteria concentration. The LOD was calculated as three times the standard deviation of the blank (measured as the R_ct_ of the sensor in the absence of *S. epidermidis*, *n* = 3) divided by the slope of the linear regression curve (y = 1034.8x + 139.72, R² = 0.9986), yielding an LOD of approximately 1 CFU/mL. This value was experimentally verified as the lowest concentration producing a signal reliably distinguishable from the blank, with measurements conducted using a µStat-i 400 analyzer (Metrohm Inula GmbH).

### 2.8. Statistical Analysis

Statistical analysis was conducted using analysis of variance (ANOVA), followed by an independent *t*-test to compare the mean surface roughness between bacteria incubated with and without MIP nanoparticles. Surface roughness was measured using Gwyddion software version 3.0. A difference was considered statistically significant at a *p*-value < 0.005 and non-significant at *p* > 0.005.

## 3. Results and Discussion

### 3.1. Isolation and Testing of S. epidermidis

**Figure 4:**
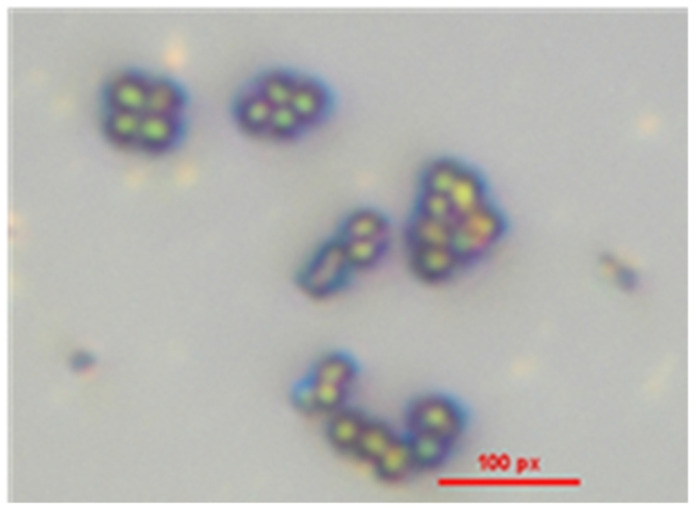
Microscopic analysis of Gram-positive *S. epidermidis*.

Figure 4 presents a microscopic image of the *S*. *epidermidis* stock culture, demonstrating the absence of contamination by other microorganisms. Gram staining was employed to confirm the presence of Gram-positive bacteria, revealing exclusively crystal violet-stained cells under microscopic examination. The analysis identified *S*. *epidermidis* as Gram-positive cocci, characterized by spherical cells arranged in grape-like clusters. Additionally, no evidence of spore formation was observed, further confirming the morphological characteristics typical of *S*. *epidermidis*.

### 3.2. Characterization of Synthesized nanoMIPs

**Figure 5:**
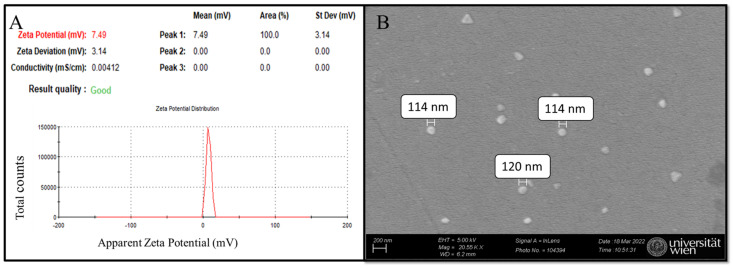
nanoMIPs characterization: (**A**) DLS analysis and (**B**) SEM micrograph of synthesized nanoMIPs.

Dynamic light scattering (DLS) of nanoMIP samples in distilled water (pH ≈ 7) reveals a zeta potential of 7.49 mV, indicating a near-neutral surface charge and suggesting stability with minimal aggregation (Figure 5A) [35,36]. For particle size determination, scanning electron microscopy (SEM) was employed, confirming that the synthesized nanoMIPs are globular, with approximate diameters ranging from 114 to 120 nm based on manual measurements of a limited number of particles in the micrograph (Figure 5B) [35]. The selection of monomer composition (composition 3 from a previous study) was primarily based on its non-negative zeta potential values, which is consistent with previous research that was measured to be close to zero [37]. This criterion was chosen to ensure stability and minimize aggregation, as zeta potential values near zero often indicate a balance between repulsive and attractive forces among particles, which is critical for colloidal stability [37]. Furthermore, the zeta potential highly depends on the solvent environment, particularly pH, ref. [38]. Hence, the synthesized nanoMIPs are consistent with previous research [35] and are suitable for further electrochemical experiments. While the majority of nanoMIPs observed in the SEM micrographs (Figure 5B) are globular, a minor fraction of particles appears triangular. This variation in morphology may arise from statistical variations during free radical polymerization, though it does not appear to compromise the overall homogeneity and functionality of the nanoMIPs: elution relies on binding strength between the imprinted cavity and the template [35].

### 3.3. Determining the Optimal Time for Forming Self-Assembled Monolayers (SAM)

**Figure 6:**
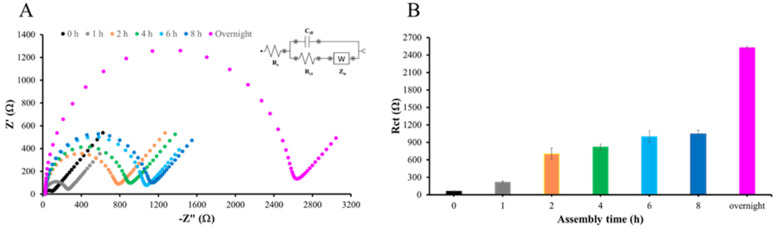
SAM formation of MHDA: (**A**) Nyquist plot of EIS measurements for AuSPE immersed in 5 mM MHDA at various time intervals and (**B**) R_ct_ values at various time intervals, with overnight corresponding to approximately 12 h of immersion in 5 mM MHDA solution at room temperature.

Figure 6A presents the impedance data in the form of a Nyquist plot, demonstrating that overnight immersion of AuSPE in 5 mM MHDA solution results in the highest resistance. The formation of the MHDA self-assembled monolayer (SAM) begins with the chemisorption of thiol groups onto the gold surface, a process driven by thermodynamics. This step is critical as it establishes the foundational layer for subsequent modifications. The strong affinity between thiol groups and gold surfaces is well documented, ensuring a stable and ordered monolayer formation [39,40]. Calculations of R_ct_ values at each time point, performed using DropView software, also confirmed that overnight immersion produced the maximum charge transfer resistance and, hence, the densest monolayer on the electrode surface. Therefore, overnight immersion of AuSPE in 5 mM MHDA solution was selected to form a SAM throughout further experiments (Figure 6B).

### 3.4. nanoMIP-Based AuSPE Fabrication

**Figure 7:**
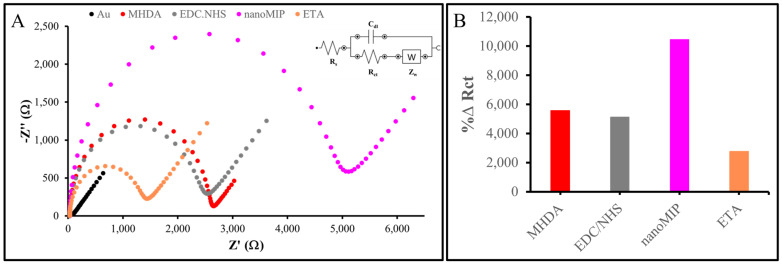
Characterization of nanoMIP-based sensor: (**A**) Nyquist plot of nanoMIP-based AuSPE. (**B**) %∆ R_ct_ value of each fabrication step detected by EIS measurement.

The nanoMIPs were functionalized with primary amino groups in order to covalently attach them to the gold surface of the sensor. Gold electrodes were cleaned and then functionalized by directly attaching the nanoMIPs onto the gold sensor chip via amine coupling. The representative EIS spectra are presented in Figure 7A, with impedance values recorded as Nyquist plots. The bare AuSPE showed the lowest R_ct_, which increased following the formation of a SAM of MHDA on the electrode surface, due to the thermodynamically favored chemisorption of thiol groups onto the gold substrate [41]. Furthermore, at neutral to basic pH levels, deprotonation of the SAM interfacial carboxylic acid groups occurs. This causes electrostatic repulsion between the negatively charged interface and the anionic redox probe, resulting in an elevated R_ct_ value compared to the bare electrode signal [42]. Subsequent to the immobilization of MHDA on the sensor surface, the carboxylic groups were activated using EDC/NHS. This activation resulted in a reduction in the negative charges within the SAM, consequently leading to a decrease in the R_ct_ value [43]. The activated carboxylic groups reacted with the primary amino groups of the nanoMIPs, thereby facilitating their covalent attachment. Consequently, the R_ct_ value increased once again upon nanoMIP attachment, which can be attributed to the nanoMIPs’ size and insulating properties. ETA was employed to block any unreacted and activated carboxylic groups, thereby reducing non-specific binding. By interacting with the unreacted MHDA, ethanolamine decreased the negative charges and introduced hydrophilic groups, resulting in a lower R_ct_ value. The EIS data indicated successful attachment of the nanoMIPs to the electrode surface, although the possibility of nanoMIPs adsorption onto the surface cannot be entirely ruled out. In terms of %∆R_ct_, with bare AuSPE as the reference, these values changed at each fabrication stage, particularly during nanoMIP attachment, confirming the successful construction of the nanoMIP-based sensor (Figure 7B).

**Figure 8:**
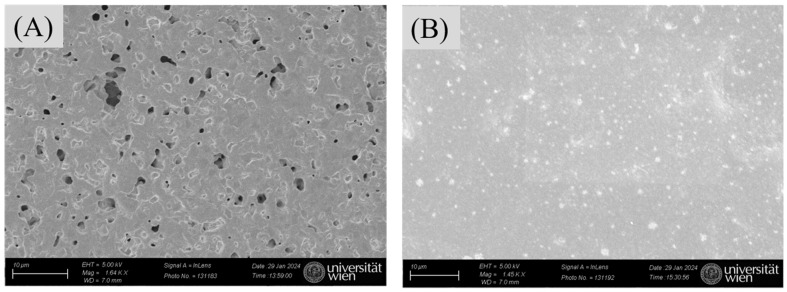
SEM analysis: bare AuSPE surfaces (**A**) and the surface of the nanoMIP-based sensor (**B**).

**Figure 9:**
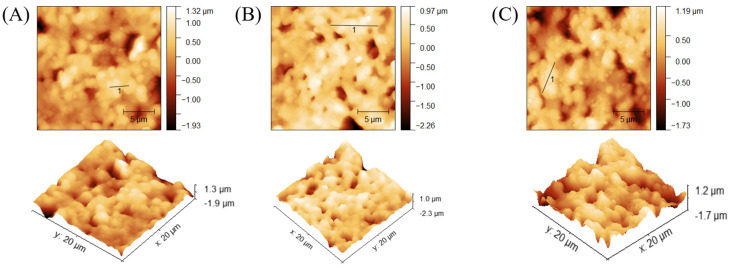
AFM analysis of different surfaces: bare AuSPE (**A**), nanoMIP-based sensor (**B**), and *S. epidermidis*/nanoMIP-based sensor (**C**).

SEM imaging served to confirm successful attachment of nanoMIPs to the modified AuSPE by comparing the surfaces of bare AuSPE and the nanoMIP-based sensors. Figure 8A indicates that the commercial bare AuSPE surface appears relatively rough and porous, whereas the modified AuSPE shows nanoMIP particles distributed across the surface. This observation confirms successful immobilization of nanoMIPs onto the AuSPE electrode (Figure 8B). In addition to verifying nanoMIP attachment via SEM, the nanoMIP-based sensor was assessed by atomic force microscopy (AFM) to confirm successful surface modification and bacterial binding. The average surface roughness (Ra) and average surface height (Ha) provide quantitative evidence of the progressive buildup of the sensor surface: Ra increased from 26.22 ± 1.5 nm for bare AuSPE to 80.86 ± 1.5 nm after nanoMIP immobilization, and further to 130.7 ± 1.5 nm upon *S*. *epidermidis* binding (Table 1, Figure 9). Similarly, Ha values rose from 180 ± 0.5 nm to 604.69 ± 0.5 nm and 783.72 ± 0.5 nm, respectively. These increases in roughness and height validate the attachment of nanoMIPs and the specific capture of *S*. *epidermidis*, demonstrating the sensor’s functionality at a nanoscale level.

### 3.5. Selectivity

**Figure 10:**
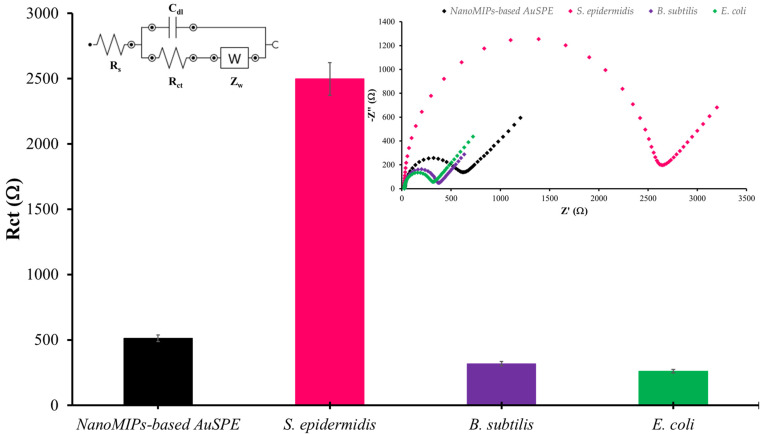
The selectivity tests for each bacterial strain at the same concentration of 10^1^ CFU/mL using 1X PBS, pH 7.4 as diluent solution. (Inset shows Nyquist plots representing the selectivity evaluation of the nanoMIP-based sensor).

Selectivity is a key issue for any sensor. To assess it for the nanoMIP-based AuSPE detecting *S*. *epidermidis*, both Gram-positive bacteria (*B. subtilis*) and Gram-negative ones (*E. coli*) were evaluated in the same way as *S. epidermidis*. The Nyquist plots revealed that *S. epidermidis* exhibits the highest impedance (Figure 10). The changes in electrochemical response caused by non-specific binding of competing bacteria were significantly lower than the response induced by the specific binding of *S. epidermidis*, they were in the baseline range of R_ct_. The results indicate that the nanoMIP-based sensor demonstrates specific binding to the target bacteria. The selectivity of MIPs for *Staphylococcus* species has been extensively explored, demonstrating significant potential in targeted detection and sensing applications. MIPs possess the ability to specifically recognize and bind *Staphylococcus* species through complementary interactions, making them highly selective [44]. MIPs can be tailored to selectively bind to specific bacterial surface on *Staphylococcus* sp. through the precise arrangement of functional groups within the polymer matrix. This selectivity is achieved by polymerizing monomers in the presence of the target functional group, creating highly selective binding sites that can effectively differentiate the target functional group from other substances. This study underscores the potential of MIPs in developing highly selective and sensitive sensors for detecting bacterial surface functional group, which could be valuable in medical diagnostics and environmental monitoring [45]. When these nanoMIPs serve as receptors on AuSPE and tested for specificity through the measurement of electrical response using EIS mode, a distinct signal change is observed for *S. epidermidis*. Consequently, the proposed platform exhibits robust performance for the detection of *S. epidermidis*. Additionally, this method is highly adaptable for detecting other species, such as the *S*. *aureus*, because selectivity depends on the template species.

### 3.6. Sensitivity

**Figure 11:**
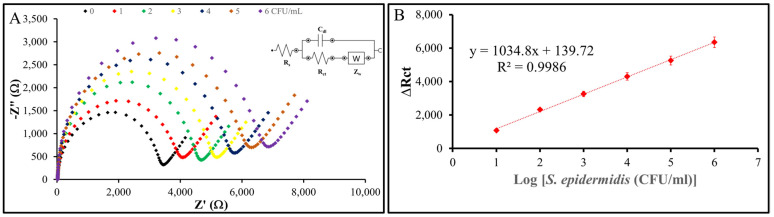
Sensitivity of the sensor system. (**A**) Nyquist plots of the nanoMIP-based sensor after incubation with varying concentrations of S. epidermidis demonstrate the sensor’s impedimetric response. (**B**) The plots illustrate the relationship between the impedance response (ΔR_ct_) of the nanoMIP-based sensor and the different concentrations of S. epidermidis (*n* = 3). The calibration curve exhibited a clear linear regression, described by the equation y = 1034.8x + 139.72, with an R² value of 0.9986. The sensor achieved a detection limit of 1 CFU/mL and a linear detection range of 10^1^–10⁶ CFU/mL for S. epidermidis.

To evaluate the sensitivity and limit of detection (LOD) of the nanoMIP-based sensor, the modified electrode was exposed to *S. epidermidis* samples across a range of concentrations down to 1 CFU/mL. Figure 11 shows the results: the change in charge transfer resistance (ΔR_ct_) is the difference between the R_ct_ value after incubation with *S. epidermidis* and the baseline R_ct_ value of the nanoMIP-modified AuSPE electrode without bacteria (ΔR_ct_ = R_ct_ (after incubation) − R_ct_ (baseline)). EIS measurements were performed in triplicate (*n* = 3) for each concentration, with standard deviations of ΔR_ct_ consistently below 5% of the mean values, demonstrating high reproducibility. These standard deviations were used to determine the LOD using the formula LOD = 3σ/S, where σ represents the standard deviation of the blank response (derived from three replicates of the baseline electrode) and S is the slope of the calibration curve obtained from the linear regression of ΔR_ct_ versus the decadic logarithm of *S. epidermidis* concentration. The calculated R_ct_ values, when plotted against the logarithmic bacterial concentration, exhibit a clear linear trend with an R² value of 0.9986, confirming the reliability of the sensor. These results indicate that the nanoMIP-based sensor developed in this study is capable of detecting *S. epidermidis* at concentrations as low as 1 CFU/mL, highlighting its exceptional sensitivity and potential for practical applications.

*Staphylococcus epidermidis* was employed as a model organism for methicillin-resistant *Staphylococcus aureus* (MRSA) [46]. Although *S. epidermidis* is classified as a biosafety level 1 (BSL-1) microorganism, certain strains are recognized as a source of nosocomial infections, particularly through the formation of biofilms on implanted plastic medical devices such as dialysis catheters [47]. Consequently, its limit of detection (LOD) plays as key factor for significant medical relevance. Certain performance characteristics of the fabricated sensor were compared with those of other *Staphylococcus* sp. sensors relying on different methods for the detection (Table 2). Among the sensors evaluated, the nanoMIP-based sensor developed in our study demonstrated the lowest limit of detection. This performance was surpassed only by the electrochemical amperometric aptasensor reported by Abbaspour et al. [48] and the electrochemical impedimetric aptasensor by Ranjbar & Shahrokhian [49], which exhibited similar results as this work. The works by Sunagar et al. [50] and Kim et al. [51] involved the use of basic amplification technique to detect *S. epidermidis*. However, there were some drawbacks involving the limitation on requiring expensive instruments, the operation is highly specialized and requires special operation training, and intolerance to some substances [52,53]. The work by Wang et al. [54] involved a more advanced technique using recombinase polymerase amplification and lateral flow strips (RPA-LFS). While this offers notable advantages, including isothermal amplification, rapid processing, minimal operational and instrumentation requirements, resistance to inhibitors, and tolerance for mismatches, several limitations must also be addressed. These include the high cost of reagents, the limited availability of commercial kits, which restricts large-scale detection, the absence of specialized primer design software, its confinement to scientific research rather than clinical applications, susceptibility to non-specific amplification, and suboptimal quantitative resolution [52,53,55,56]. Xia et al. [57] that utilized piezoelectric quartz crystal microbalance (QCM) biosensors employing nucleic acids as biomolecules, which provides notable advantages, including simplicity and cost-effectiveness, positioning them as valuable tools in molecular analysis [58,59,60,61]. Similarly, gold-modified extended gate field-effect transistor (Au-EGFET) sensors, while primarily developed for biofilm detection performed by Purwidyantri et al. [62], have shown significant potential in detecting specific bacterial species associated with infections through whole-gate surface modification. Nonetheless, both approaches are limited by low sensitivity, necessitating relatively high bacterial concentrations for *S*. *epidermidis*. The nanoMIP-based sensor, in this work, yields a low limit of detection equal previous work [48,49]. Even though this work uses *S*. *epidermidis* as a representative *Staphylococcus* spp., it shows a big improvement in bacterial detection technology by suggesting that Table 2 compares published works of electrochemical sensors using nanoMIPs to detect *S*. *epidermidis*. Our new developed sensor, which uses nanoMIPs and EIS, can detect bacteria at an incredibly low level of 1 CFU/mL and covers a wide range of 10¹–10⁶ CFU/mL. This is much better than existing sensors, like the cell-imprinted polymer (CIP)-based EIS sensor, which can only detect down to 10³ CFU/mL as shown in Table 2. By comparing our novel sensor with other MIP-based approaches in the literature, Table 2 can highlight our sensor’s novelty and superiority. Our sensor uses nanoMIPs with AuSPE, offering high sensitivity, selectivity, and cost-effectiveness. This comparison underscores this study’s important contribution to rapid, reliable detection of *S*. *epidermidis*, a common hospital-acquired infection associated with biofilms on medical devices. This work marks a significant advancement in clinical diagnostics and infection control capable of beneficial for *Staphylococcus* monitoring and control.

## 4. Conclusions

In this study, a nanoMIP-based AuSPE sensor was developed, demonstrating exceptional sensitivity and selectivity for detecting *Staphylococcus epidermidis*, with a detection limit of 1 CFU/mL and a linear range of 10¹–10⁶ CFU/mL. This performance surpasses conventional methods such as PCR and real-time PCR, as well as other MIP-based sensors, owing to the innovative integration of solid-phase synthesized nanoMIPs with electrochemical impedance spectroscopy (EIS). The use of whole *S*. *epidermidis* as a template for nanoMIP synthesis represents a novel approach, enabling specific recognition of bacterial surface features, as confirmed by selectivity tests against *Escherichia coli* and *Bacillus subtilis*. The sensor’s rapid analysis time (<30 min), cost-effectiveness, and disposability further distinguish it from existing technologies, offering a practical solution for real-time monitoring of biofilm-associated infections on medical devices. Characterization via DLS, SEM, and AFM underscores the robustness of the fabrication process, with nanoscale surface changes validating the sensor’s functionality. This work advances bacterial detection technology by combining synthetic receptors with electrochemical sensing, addressing critical needs in clinical diagnostics and infection control. Future research will focus on validating the sensor in complex biological matrices, such as clinical samples, and adapting the platform for other pathogens, such as *S*. *aureus*. Additionally, integrating this sensor into medical device coatings or portable diagnostic systems could enhance its utility in healthcare settings, paving the way for broader applications in infection prevention and management.

## Figures and Tables

**Table 1:** Analysis results of average surface roughness (Ra) and average surface height (Ha).

Surface	AuSPE	nanoMIP Sensor	*S. epidermidis*/nanoMIP Sensor
Roughness (Ra, nm)	26.22 ± 1.5	80.86 ± 1.5	130.7 ± 1.5
Height (Ha, nm)	180 ± 0.5	604.69 ± 0.5	783.72 ± 0.5

**Table 2:** Comparison of the performance characteristics of the nanoMIP-based sensor developed in this work with relevant sensors for S. epidermidis.

Detection Method	Detection Range (CFU/mL)	Detection Limit (CFU/mL)	Ref.
PCR	-	10^6^	[50]
Real-time PCR	10^2^–10^8^	2.6 × 10^2^	[51]
Recombinase polymerase amplification and lateral flow strips (RPA-LFS)	8 × 10^−1^–8 × 10^5^	8.91	[54]
Piezoelectric QCM nucleic acid biosensor	1.3 × 10^3^–1.3 × 10^7^	1.3 × 10^3^	[57]
Au-EGFET sensor	3.8 × 10^6^–3.8 × 10^8^	9 × 10^5^	[62]
Cell-imprinted polymers (CIPs)-based EIS sensor	10^3^–10^7^	10^3^	[63]
nanoMIP-based EIS sensor	10^1^–10^6^	1	This study

## Data Availability

Data are contained within the article.
